# 22^nd^ AMN Congress in Bangkok, Thailand – Interview with Prof. Johannes Vester

**DOI:** 10.25122/jml-2025-1005

**Published:** 2025-10

**Authors:** Alexandra Gherman, Stefana-Andrada Dobran

**Affiliations:** 1 RoNeuro Institute for Neurological Research and Diagnostic, Cluj-Napoca, Romania; 2 Sociology Department, Babes-Bolyai University, Cluj-Napoca, Romania

**Interviewee: Professor Johannes Vester**

**Interviewer: Alexandra Gherman**

**A.G.: Dear Professor Johannes Vester, the Academy for Multidisciplinary Neurotraumatology (AMN) has reached the 22^nd^ edition of its annual congress, taking place in a very special location - Bangkok, Thailand. Please share your overall impression of this educational event and let us know if it meets your expectations**.

J.V.: Thank you for this question. I think we all realized here again the importance of face-to-face communication – to see each other, to look into our eyes and between so many different disciplines. It's important to connect to really move things forward and use the available synergies. That was fantastic during these days, so important. ‘*Think AMN*’ - that was the common experience; this interdisciplinary sharing of different scientific perspectives in an open, respectful way, as we do it here, this is a unique approach. And you see how this opens the hearts, the passion, the energy, which we all need to move things forward, because it makes sense to think that way. The living feedback cycle between all of us, between all the disciplines involved in neurotrauma, that's opening our mindset to a broader understanding of the really complex nature of neurotrauma. That's complex, especially traumatic brain injury. Also for developing successful new treatment concepts, that's only possible by putting all these different aspects together to a holistic approach. That's ‘*Think AMN!*’


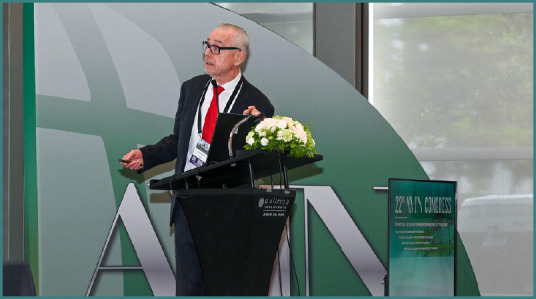


**A.G.: In your capacity as President of the Academy, what do you consider to be your best accomplishments of your term?**

J.V.: Good question! There are several of them. Well, for me personally, it's the further evolution of the AMN from a multidisciplinary starting base at that time when it was founded, to a multidimensional society. In medicine there's no truth carved in stone. Medical state-of-the-art is a snapshot. It changes with every new piece of research, of deeper understanding of the complex interdependencies. We always have to stay open to learn from each other, to stay flexible in our mindset for new developments. And, very important, neurotrauma needs not only a multidisciplinary approach, it needs a multidimensional approach – because neurotrauma is multidimensional from its very nature.


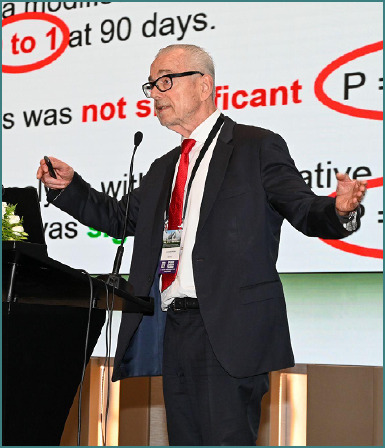


**A.G..: Can you please explain the difference?**

J.V.: Yes. What's the difference? A multidisciplinary approach refers to the involvement of professionals from multi-disciplines who work collaboratively, often independently, to treat the patient. Each specialist contributes [with] his own expertise - neurosurgeon, neurologist, neuropsychologist, physical therapist, speech therapist, psychiatrist, and so on. So, it's discipline-oriented. A multi-dimensional approach is patient-centered. That's something different. It refers to the evaluation or treatment of a condition across multiple domains or dimensions of functioning. So, it focuses simultaneously on the various dimensions of the patient's conditions, like motor, cognition, emotion, communication, social integration. All these aspects [of the] quality of life, we have to see at the same time to find the right approaches. So, we don't concentrate on our discipline, we offer our discipline for this holistic approach, but our heart is patient-centered – that makes the big difference between multidisciplinary, multi-dimensional. AMN as the Academy for Multidisciplinary Neurotraumatology is dedicated to open the mindsets to this enlarged approach to communicate the breadth of multiple perspectives simultaneously [that is] so necessary to capture the whole picture of neurotrauma and recovery. And that's really new this way and it was also extremely appreciated by the participants in this Congress, that's ‘*Think AMN*’.


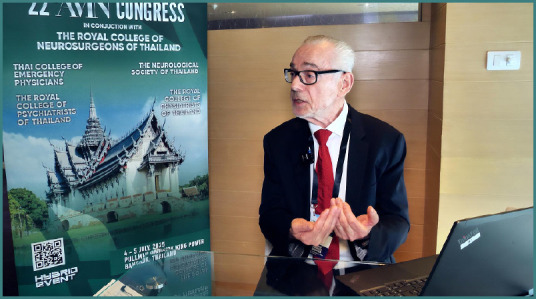


**A.G.: This year's Congress brings forward several developments pertaining to the new vision and mission of the Academy for Multidisciplinary Neurotraumatology. Could you elaborate on future perspectives related to those?**

J.V.: There are some important new developments. And one of the most important is the establishment of the AMN Focus Groups (FGs). The AMN is like a center, like a brain, like a quantum energy putting all these things together. But why? It's for the patient there, and he is locally in a certain country, in a certain hospital, with certain people working there. So, the Focus Groups are committed to implement this in a certain country, because they deal with the local constraints, with the local conditions. They have to be creative [about] how to implement ‘*Think AMN*’ in this country and this can be a different challenge than implementing “*Think AMN*” in another country. Moreover, the feedback from these focus groups will help each other because they develop, e.g., some fantastic ideas on how to deal with governmental support and that can help other countries. So, again, that's the focus now on the patient, which is, by very nature, in a certain location. The AMN Focus Groups are even one step further on to establish that ‘*Think AMN*’ in real life, in real world situations, and to learn from each other in these countries. I'm very happy that we already have six countries who said ‘*We want to enter this, we want to contribute, we want to implement the AMN thinking in our country with this spirit and implement all this for the benefit of future patients’*. It's a great success that we already have this dedication because it's also work, you know, to follow the spirit, to implement, to have ideas and to share these ideas with other countries and other focus groups. So, it's a big step forward for implementing all this.


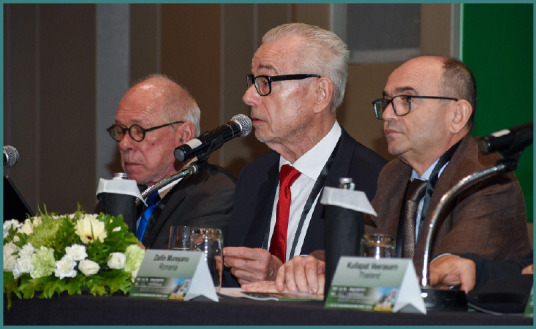


**A.G.: Indeed, it is a very good achievement and the Congress is not over, so, hopefully, there will be more Focus Groups to be announced by the end of the event. Finally, Professor Vester, as a highly acclaimed expert in biometrics, please share with us the essence of the multidimensional methodology in evaluating international clinical trials**.

J.V.: Thank you for these questions and I will tell you the essence. It is exactly related to what I said before - the multi-dimensional approach - if you want to capture the whole picture of the development of the recovery of a patient after neurotrauma - cognitive, emotional, physical, and many many different aspects, we need the statistical procedures to deal with this, to also have simultaneous procedures and, if I look back decades ago, there was only one outcome that was the Glasgow outcome scale split into two pieces: favourable outcome or unfavorable outcomes. So, every patient was just considered as favorable or not favorable. There was nothing in between. That's like looking only in black and white, in good and bad. No differentiation by this. What a different world it is to capture the full scales in cognitive, physical, emotional, social recovery and at the same time to capture the full picture of the outcome of the patient and the statistical procedures for this fortunately are available. The big breakthrough was in 2010 with the IMPACT recommendations that we cannot continue research like this - with the statistical old procedures, just the good and bad, black and white. We have to have multi - dimensional procedures and the European Medicine Agency made workshops on this, that we need simultaneous correlation - sensitive spread of outcome to capture the breadth of neuro recovery. The International Biometric Society made teaching courses. I was participating to teach other statisticians on these procedures - just to name the gold standard which is called the *Wei-Lachin procedure* which works with full scales simultaneously providing the test results as we expect for evidence-based medicine. So, there is a big breakthrough from a statistical point of view and, also, the AMN is encouraging this, we make training on this because, otherwise, we have nice ideas, but we cannot implement them in evidence-based research. Now it's happening and it is already implemented successfully; just to mention the Captain series of trials which showed the immense power of this new approach.

**A.G.: Thank you very much and I wish you all the best in all your endeavours!**

J.V.: Thank you very much! It was my pleasure!

